# Seborrhœic Dermatitis

**Published:** 1905-06-17

**Authors:** 


### SEBORRHŒIC DERMATITIS.

This, one of the commonest of all skin diseases, was originally named by Unna "Seborrhœic Eczema." It has for long been regarded as having a close connection with alopecia, and, though generally credited with a microbic origin, has not yet definitely established its microbic associations. Dr. Duncan Bulkeley[1] describes it as a lesion probably microbic, exhibiting varying degrees of scaliness, generally greasy, and having a tendency to spread by the coalescence of well-defined and commonly circular areas. It is usually primary on the scalp.

Whether or not seborrhœic dermatitis is causally connected with baldness, it is at least very frequently associated with this condition. Of 777 collected cases in Dr. Bulkeley's series, 284, or 36·5 per cent., presented well-marked baldness. He considers it definitely contagious, but not very actively so. The lesions vary much in appearance, and may simulate any of the following conditions:—Psoriasis, ringworm, tinea versicolor, pityriasis rosea, or the secondary eruptions of syphilis.

As to treatment, the author states that there is no internal specific for the disease, but that good general hygiene must not be neglected. Of local applications he recommends for the scalp a lotion prescribed as follows:—

| | |
|---|---|
| ℞ Resorcin ... ... ... | 1 to 2 drachms. |
| Spt. Vin. Rect.... ... ... | 2 to 4 drachms. |
| Glycerin ... ... ... | 2 to 4 drachms. |
| Aq. Rosæ ... ... ... | to 4 ozs. |

This lotion should be well rubbed in night and morning. It is well to remember that if the resorcin be too strong it may produce a scaliness of its own which, however, is no bar to success. For regions other than the scalp the following ointment is recommended:—

| | |
|---|---|
| ℞ Resorcin ... ... ... | 20 to 60 grains. |
| Precipitated Sulphur ... ... | 20 to 60 grains. |
| Ung. Aq. Rosæ ... ... ... | 1 oz. |

Sometimes, where these preparations do not succeed, white precipitate ointment, diluted about three times, will secure a good result. The author is strongly of opinion that such drastic applications as chrysarobin are not necessary, nor are they so successful as sulphur and resorcin, which he considers to be the mainstays of proper treatment.

[1] Med. Rec., New York, May 13, 1905.

